# *Escherichia coli* Producing CTX-M-2 β-Lactamase in Cattle, Japan

**DOI:** 10.3201/eid1001.030219

**Published:** 2004-01

**Authors:** Yutaka Shiraki, Naohiro Shibata, Yohei Doi, Yoshichika Arakawa

**Affiliations:** * Gifu Prefectural Office of Meat Inspection, Gifu, Japan; † National Institute of Infectious Diseases, Tokyo, Japan

**Keywords:** CTX-M-type beta-lactamase, extended-spectrum beta-lactamase, *Escherichia coli*, random amplified polymorphic DNA, cattle, livestock, slaughterhouse, antimicrobial-resistance, surveillance

## Abstract

From November 2000 to June 2001, *Escherichia coli* strains producing CTX-M-2 β-lactamase were isolated from 6 (1.5%) of 396 cattle fecal samples and 2 (0.7%) of 270 surface swabs of cattle carcasses in Japan. The *bla*_CTX-M-2_ gene responsible for CTX-M-2 production was encoded on transferable plasmids, and the gene was transferred to *E. coli* CSH2 with a very high frequency (2 x 10^-4^ to 6 x 10^-1^ per donor cells) by conjugation. Random amplified polymorphic DNA analysis of nine isolates showed at least five different patterns. These findings suggest that CTX-M-2 producers might have originated from cattle through the use of cephalosporins such as ceftiofur and that cattle could be a reservoir of CTX-M-2–producing *E. coli*. Continuous and strategic surveillance of antimicrobial-resistant bacteria in livestock is essential to suppress further dissemination of these bacteria into society at large.

Shortly after a variety of expanded-spectrum cephalosporins were introduced in the 1980s, bacterial strains producing extended-spectrum β-lactamases (ESBLs), such as TEM- or SHV-derived ESBLs, emerged in Europe (*1*), and since then, their variants have been proliferating around the world (*2*,*3*). More recently, CTX-M-type β-lactamases, a small but growing family of broad-spectrum class A β-lactamases, were initially discovered as MEN-1 (EMBL accession no. X92506) and also later found as Toho-1 in Japan in 1993 (*4*). Since the early 1990s, these β-lactamases have been identified in various bacterial species belonging to the family *Enterobacteriaceae* (*5*). Several questions regarding the origin and mode of proliferation of the CTX-M-type β-lactamases are unclear. Unlike TEM- and SHV-derived ESBL producers, the CTX-M-type β–lactamase producers have been incidentally and sporadically detected as single clinical isolates from patients with urinary tract infections and the like (*6*,*7*) over an extensive geographic area, including Europe, South America, and the Middle and Far East. The cause of this global distribution is not well known (*5*,*8*). Moreover, derivation of the CTX-M-type enzymes or the prototype of this enzyme with its narrow spectrum remains unknown (*9*–*12*).

In Japan, clinical isolation of the TEM- or SHV-derived ESBL producers is still rare (*13*,*14*); *Escherichia coli* strains producing CTX-M-2 β-lactamase, one of the CTX-M family, have been predominantly isolated to date (*13*). On the other hand, clinical isolates producing IMP-1 type metallo-β-lactamase, which show resistance to carbapenems and cephamycins as well as various expanded-spectrum cephalosporins, have been identified in Japan (*15*), and the proliferation of these strains has become a clinical concern (*16*). As for the disproportionately low isolation rate of the TEM- or SHV-derived ESBL producers in Japan, carbapenems and cephamycins, whose use has been restricted in many Western countries, have been preferentially used as first-line drugs in Japan (*13*,*15*). This practice makes it more plausible that TEM- or SHV-derived ESBL producers would be rarely isolated and that metallo-β-lactamases would be isolated often in Japan. However, it is not easy to explain the predominant isolation of *E. coli–*producing CTX-M-2 β-lactamase that is usually susceptible to carbapenems and cephamycins like TEM- or SHV-derived ESBL producers. In addition, since CTX-M-2 β-lactamase producers tend to be isolated from patients who have neither received antimicrobial drugs nor been hospitalized, the existence of healthy carriers of CTX-M-2 producers was suspected (*17*,*18*). Still, one cannot assume healthy carriers exist on the basis of the low isolation rate of strains producing broad-spectrum class A β-lactamases in Japan.

One hypothesis to address these issues is that CTX-M-2 might emerge elsewhere than in humans and that the enzyme might have originated in livestock. Recently, a global threat developed because certain antimicrobial-resistant bacteria, such as vancomycin-resistant enterococci (*19*), *Salmonella enterica* Typhimurium DT104 (*20*) and fluoroquinolone-resistant *Campylobacter jejuni and C. coli* (*21*) emerged in food animals possibly through the use of antimicrobial drugs for growth promotion or disease treatment. However, few reports have been published about strains in animals producing ESBLs or CMY-type cephamycinases, which confer resistance to expanded-spectrum cephalosporins (*22*,*23*), and no CTX-M-type β-lactamase producer has been isolated from animals. Therefore, to examine this hypothesis, we conducted a study to isolate any strains producing extended-spectrum class A β-lactamases from cattle at Japanese slaughterhouses.

## Materials and Methods

### Sampling and Bacterial Culture

From November 2000 to June 2001, a total of 396 fecal samples of cattle and surface swabs of 270 cattle carcasses were collected at two slaughterhouses in Gifu Prefecture, Japan. ESBL screening agar plates (*17*), which were prepared using BTB Lactose agar (Nissui Pharmaceutical Co., Tokyo, Japan) containing 2 μg/mL of cefotaxime (Chugai Pharmaceutical Co., Ltd., Tokyo, Japan) and 8 μg/mL of vancomycin (Shionogi & Pharmaceutical Co., Osaka, Japan), were used to isolate gram-negative enterobacteria that produce broad-spectrum class A β-lactamases. One swab was used to sample each cattle feces, and two swabs were used for each cattle carcass. For sampling of the cattle feces, a swab was inserted into the core of a lump of feces. When several cattle were kept in the same enclosure, a direct rectal swab was sampled from each of the cattle. Shoulder and rump were swabbed separately in each cattle carcass; the size of the swabbed area was approximately 20 x 20-square centimeters for each swab. Swabs of feces were plated directly on the screening agar. Swabs of carcasses were suspended in a 10-mL Trypticase soy broth (Nissui Pharmaceutical Co.) containing 2 μg/mL of cefotaxime and 8 μg/mL of vancomycin, and then plated on the screening agar. The remaining Trypticase soy broth with bacteria was further incubated overnight. A swab of bacterial culture was then plated on the screening agar. Colonies suspected to be enterobacteria were isolated and identified by using the API 20E system (bioMérieux, Marcy l’Etoile, France). *E. coli* isolates were serotyped with a slide agglutination kit (Denka Seiken Co., Ltd., Tokyo, Japan) and were screened for genes of virulence factors, including Shiga toxins and *E. coli* attaching and effacing factor by polymerase chain reaction (PCR) (*24*).

### Detection of β-Lactamases

The acidmetric β-lactamase test was performed by using P/Case TEST (Showa Yakuhin Kako Co., Ltd., Tokyo, Japan) to detect β-lactamase production in the isolates. According to the manufacturer’s instructions, the colonies were spread on two indicator disks, containing benzylpenicillin and cephaloridine with clavulanic acid, respectively. When the strain produces class A β-lactamases, including TEM- or SHV-derived ESBLs, or CTX-M-type enzymes, the color of a disk containing benzylpenicillin turns yellow. The other disk, containing cephaloridine with clavulanic acid, remains purple because hydrolysis of cephaloridine by the class A β-lactamases is blocked in the presence of clavulanic acid. If the strain produces class C or class B β-lactamases, both disks turn yellow because these enzymes are no longer blocked by clavulanic acid. The isolates suggested to produce extended-spectrum class A β-lactamase were further investigated to determine whether they produced ESBLs by the double-disk diffusion test (*25*), using two Kirby-Bauer disks (Eiken Chemical Co., Ltd., Tokyo, Japan). A swab of bacterial culture (approximately 10^6^ CFU/mL) to be tested was spread on a Mueller-Hinton agar plate (Eiken Chemical Co.), and one disk containing cefotaxime, ceftazidime, ceftriaxone, cefpodoxime, aztreonam, or cefepime was put on the plate. The other disk, containing amoxicillin+clavulanic acid, was also placed alongside the first disk (center-to-center distance of approximately 3 cm), and the agar plate was then incubated for 18 hours. When an expansion of the inhibitory zone between the two disks was observed, the isolates were speculated to produce ESBL.

### Conjugation and Plasmid Profiles

Conjugation experiments were performed by using *E. coli* CSH2 as a recipient, as previously described (*17*). A mixture of donor and recipient strains was incubated in Luria-Bertani broth (Difco Laboratories, Detroit, MI) at 37°C for 18 hours. Transconjugants were selected by using BTB Lactose agar plates supplemented with 100 μg/mL of rifampicin (Daiichi Pharmaceutical Co., Ltd., Tokyo, Japan) and 2 μg/mL of cefotaxime to inhibit the growth of the donor strain and the recipient strain, respectively. Frequency of transfer was calculated by dividing the number of transconjugants by the number of donors. Plasmid DNA was prepared from the isolates and their transconjugants by using Quantum Prep Plasmid Miniprep Kit (Bio-Rad Laboratories, Richmond, CA), according to the manufacturer’s instructions. After agarose gel electrophoresis, the sizes of the plasmids were determined by comparing their migration distances with those of plasmids of known sizes.

### Susceptibility Testing

MICs were determined by overnight broth-microdilution method using MicroScan ESBL Confirmation Panel (Dade Behring, Sacramento, CA). This panel was designed to detect ESBL producers in accordance with the National Committee for Clinical Laboratory Standards (NCCLS) document M100-S9 (*26*). The MIC of ceftiofur (Pharmacia Co., Kalamazoo, MI), an expanded-spectrum cephalosporin often used in veterinary medicine, was also determined by the broth-microdilution method in accordance with NCCLS document M7-A4 (*27*). *E. coli* ATCC 25922 and *Pseudomonas aeruginosa* ATCC 27853 were used as quality-control strains.

### PCR and DNA Sequencing

To determine the genotype of strains producing broad-spectrum class A β-lactamases, PCR was performed by using primers specific to TEM, SHV (*13*), CTX-M-1 (MEN-1) (*28*), CTX-M-2 (*29*), and CTX-M-9 (*8*) genes. The PCR products were sequenced by using a BigDye Terminator Cycle Sequencing Ready Reaction kit (Applied Biosystems, Foster City, CA) with the same primers for PCR. The DNA sequences were analyzed in an ABI PRISM 377 XL Sequencer Analyzer (Applied Biosystems).

### RAPD Analysis

Random amplified polymorphic DNA (RAPD) analysis was performed by using Ready-To-Go RAPD analysis beads (Amersham Pharmacia Biotech, Piscataway, New Jersey), according to the manufacturer’s instructions. DNA was prepared from the isolates using InstaGene DNA Purification Matrix (Bio-Rad Laboratories), also according to the manufacturer’s instructions. The reaction mixture contained 25 pmol of one of six RAPD analysis primers (Amersham Pharmacia Biotech) and 10 μL of DNA preparation in a final volume of 25 μL. Amplification was performed with initial denaturation at 95°C for 5 minutes, followed by 45 cycles of 1 minute at 95°C, 1 minute at 36°C, and 2 minutes at 72°C. The amplified products were separated by electrophoresis in 1.5% agarose gel. The fingerprints were compared visually, and patterns were considered different when they differed by at least one amplification band.

## Results

### Identification of β-Lactamases

Of 396 fecal samples of cattle, 104 (26.3%) samples gave colonies on the ESBL screening agar. Among the strains grown on the screening agar, 32 strains of *E. coli* and 2 strains of *Citrobacter koseri* were positive through the P/Case TEST for production of penicillinase, cephalosporinase, or both (Table 1). The double-disk diffusion test was performed on 28 strains that were speculated to produce penicillinase; 7 strains isolated from 6 (1.5%) of 396 fecal samples were positive. However, two strains, GS553 and GS554, which produced cephalosporinase and penicillinase, showed a clear expansion of the inhibitory zone only when a disk of cefepime, a better detection agent for ESBLs in the presence of an AmpC β-lactamase (*30*), was used. By a PCR analysis with a set of PCR primers specific for *bla*_CTX-M-2_, a 900-bp fragment was amplified from the seven strains that were positive in the double-disk diffusion test (Table 1). However, since CTX-M-2 and Toho-1 have only one amino acid substitution, genes for *bla*_CTX-M-2_ and *bla*_Toho-1_ were indistinguishable by the PCR. DNA sequencing of the PCR products subsequently showed that all were 100% identical with the *bla*_CTX-M-2_ reported (*31*). Similarly, two strains isolated from 2 (0.7%) of 270 surface swab samples of cattle carcasses were positive in the double-disk diffusion test and possessed *bla*_CTX-M-2_ (Table 1).

**Table 1:** Number of β-lactamase producers isolated from cattle^a^

Sample (no.)	Species	Acidmetric β-lactamase test				PCR typing
		Total	PC	PC and CS	CS	
Feces (396)	*Escherichia coli*	32	7	19	6	7 (CTX-M-2)
	*Citrobacter koseri*	2	2	0	0	0
Swab^b^ (270)	*E. coli*	5	2	2	1	2 (CTX-M-2)
	*C. freundii*	1	0	1	0	0

^a^PC, penicillinase; CS, cephalosporinase; PCR, polymerase chain reaction. ^b^Swab, surface swab of cattle carcass.

Although all 9 isolates producing CTX-M-2 β-lactamase were *E. coli*, their serotype of O antigen could not be defined with 43 commercially available antisera that were representative serotypes of pathogenic *E. coli.* Moreover, genes of virulence factors described previously were not detected from the strains by PCR.

### Antimicrobial Susceptibility Testing

The susceptibilities of two representative isolates, GS528 and GS554, and their transconjugants are shown in Table 2. All the isolates were resistant to piperacillin, cefotaxime, ceftriaxone, cefpodoxime, cefepime, and aztreonam, and more resistant to cefotaxime than to ceftazidime. Except for strains GS553 and GS554, the β-lactamase inhibitor clavulanic acid (fixed concentration of 4 μg/mL) reduced MICs of cefotaxime and ceftazidime by >2^10^- and 2^6^-fold, respectively. These susceptibility profiles of the isolates were similar to those observed for strains that produced CTX-M-2 β-lactamase (*31*). Both GS553 and GS554 strains, which produced cephalosporinase as well as penicillinase, were resistant to cefotetan, cefmetazole, and cefoxitin as well as piperacillin, cefotaxime, ceftriaxone, cefpodoxime, cefepime, and aztreonam. In addition, clavulanic acid hardly reduced the resistance levels of these two strains to cefotaxime and ceftazidime. These results, together with those obtained through the double-disk diffusion test, suggested that both GS553 and GS554 strains produced putative AmpC β-lactamase at high levels as well as the CTX-M-2 β-lactamase. All the isolates producing CTX-M-2 β-lactamase were highly resistant to ceftiofur (MIC >1,024 μg/mL).

**Table 2:** MICs of β-lactams for *Escherichia coli* strains isolated from cattle, transconjugants, and recipients^a^

	MIC (μg/mL) for *E. coli* strain:				
Antimicrobial drug	GS528	CSH2 trGS528	GS554	CSH2 trGS554	CSH2
Piperacillin	>64	>64	>64	>64	<16
Cefotaxime	>128	>128	>128	>128	<0.5
Cefotaxime + CLA^b^	<0.12	<0.12	32	32	<0.12
Ceftazidime	2	4	32	32	<0.5
Ceftazidime + CLA	<0.12	<0.12	16	32	<0.12
Aztreonam	>64	64	>64	64	<0.5
Ceftriaxone	>64	>64	>64	>64	<0.5
Cefpodoxime	>64	>64	>64	>64	<0.5
Cefepime	>32	>32	>32	>32	<1
Cefotetan	<0.5	<0.5	>32	>32	<0.5
Cefmetazole	1	1	>16	>16	1
Cefoxitin	<2	<2	>32	>32	<2
Meropenem	<0.5	<0.5	8	4	<0.5
Ceftiofur	>1,024	>1,024	>1,024	>1,024	<0.25

^a^*E. coli* CSH2 trGS528 and trGS554 were transconjugants of *E. coli* GS528 and GS554, respectively. ^b^CLA, clavulanic acid at a fixed concentration of 4 μg/mL.

### Plasmid and RAPD Analysis

Conjugation experiments indicated that all the isolates were able to transfer their cefotaxime resistance to the recipient and that the resistance to cephamycins observed in both strains GS553 and GS554 was also transferred to the transconjugant. All transconjugants produced the same β-lactamase(s) of their donor strains, and susceptibility profiles of the transconjugants were also similar to those of donor strains (Table 2). These results demonstrated that *bla*_CTX-M-2_ genes of the isolates might be encoded on transferable plasmids. The frequency of transfer was very high (2 x 10^-4^ to 6 x 10^-1^ per donor cells) (Table 3). Plasmid profiles of the isolates showed one to three large plasmids with five different patterns in each strain, while an approximately 33-MDa plasmid was common among all the strains. Approximately 33-MDa and 50-MDa plasmids were both transferred to recipient cells in all the strains (Table 3). RAPD analysis of a total of nine isolates gave at least five different patterns (Figure, Table 3). Although strains GS553 and GS554 were isolated from the same fecal sample, they differed in RAPD pattern and plasmid profile.

**Table 3:** Characteristics of CTX-M-2 β-lactamase-producing *Escherichia coli* isolated from cattle^a^

Strain	Source	β-lactamase^b^	Plasmid profile (MDa)	Transferred plasmid (MDa)	Frequency of transfer	RAPD pattern
GS528	Feces 1	PC	33, 50, 86	33, 50	6 x 10^-4^	A
GS542	Feces 2	PC	33, 50, 86	33, 50	2 x 10^-4^	A
GS547	Feces 3	PC	33, 50, 86	33, 50	3 x 10^-4^	A
GS553	Feces 4	PC and CS	33, 50, 61	33, 50	3 x 10^-1^	B
GS554	Feces 4	PC and CS	33, 50	33, 50	2 x 10^-1^	C
GS721	Feces 5	PC	33	33	9 x 10^-2^	D
GS733	Feces 6	PC	33	33	2 x 10^-1^	D
GS631	Swab^c^ 1	PC	33, 86	33	5 x 10^-1^	E
GS671	Swab 2	PC	33, 86	33	6 x 10^-1^	E

^a^PC, penicillinase; CS, cephalosporinase; RAPD, random amplified polymorphic DNA. ^b^β-lactamases were detected by acidmetric β-lactamase test. ^c^Swab, surface swab of cattle carcass.

**Figure:**
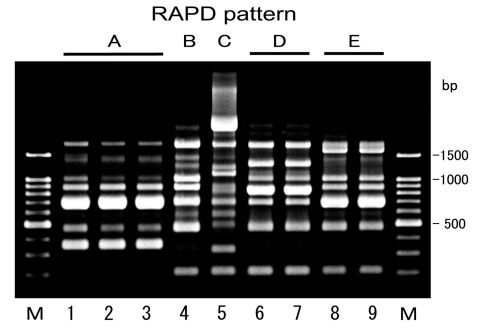
Random amplified polymorphic DNA (RAPD) patterns of CTX-M-2 β-lactamase-producing *Escherichia coli* isolated from cattle. Lanes M, 100-bp DNA ladder; lanes 1–9, strains GS528, GS542, GS547, GS553, GS554, GS721, GS733, GS631, and GS671, respectively. Five RAPD patterns, A to E, were produced with RAPD analysis primer 4 (Amersham Pharmacia Biotech, Piscataway, NJ).

## Discussion

We investigated the cause of the disproportionate emergence of CTX-M-2 β-lactamase and so-called ESBLs, including TEM- or SHV-derived enzymes, in Japan. We isolated *E. coli* strains producing CTX-M-2 β-lactamase from 6 (1.5%) of 396 fecal samples from cattle and 2 (0.7%) of 270 surface swabs of cattle carcasses. Negative results, however, do not necessarily mean the organisms are absent on the slaughterhouse carcasses because of the limited size of the overall swabbed surface area.

Our findings raised a complex question: Did CTX-M-2 β-lactamase producers initially emerge in cattle or humans? We assume they emerged from cattle. Indeed, we found no direct evidence of transmission of *E. coli* strains producing CTX-M-2 β-lactamase from cattle to humans, but our results strongly suggested that transmission of the CTX-M-2 producing microorganism might have occurred between cattle and humans. This speculation is supported by the fact that CTX-M-2 β-lactamase-producers isolated from humans in Japan are identified predominantly as *E. coli,* as was observed in Japanese cattle. According to the survey of ESBLs in human clinical isolates in Japan, Toho-1-type β-lactamase was the most prevalent, and half of the Toho-1-type β-lactamase producers were *E. coli* (*13*). Moreover, the Toho-1-type β-lactamases reported in several studies in Japan were later found to be CTX-M-2 by PCR and sequencing analyses. Furthermore, according to the survey of ESBL producers in human stool specimens reported recently in Japan, Toho-1-type β-lactamase-producing enterobacteria were isolated from 2 (0.5%) of 366 specimens (*17*). Since the survey samples were from 231 inpatients and 135 outpatients with diarrhea, the rate of CTX-M-2 producers in healthy humans in Japan is estimated to be <0.5%. Indeed, by chi-square analysis, the isolation rate (1.5%) of CTX-M-2 producers in cattle feces obtained in our study showed no statistically significant difference from that of ESBL producers in human cases reported previously in Japan (*17*). However, we speculate that CTX-M-2 producers found in cattle have something to do with those from humans. Many reports substantiate that bacteria can be transmitted from food-producing animals to humans through the food chain, and we found that the surface of cattle carcasses was stained with the CTX-M-2–producing bacteria. Our speculation is also supported by the fact that TEM- or SHV-derived ESBLs have not been detected from livestock so far even in Western countries, where TEM- or SHV-derived ESBLs have been widely detected with a high frequency in various medical institutions. In other words, if transmission of ESBL producers from human to cattle can occur with some frequency, several TEM- or SHV-derived ESBL producers would be isolated also from cattle. However, no such finding has been reported even in Western countries. Thus, prospective investigations should be conducted to understand the current status of *E. coli* strains that produce CTX-M-enzymes in livestock, especially in those countries where CTX-M-enzymes have been found in humans.

Recently, SHV-12 β-lactamase–producing *E. coli* was isolated from a dog with recurrent urinary tract infections (*22*). The origin of the isolate, however, was not known since the treatment with expanded-spectrum cephalosporins was not been recorded. In livestock, although penicillinases such as TEM-1 and TEM-2 have been identified from cattle (*23*,*32*–*34*), pigs (*35*), and poultry (*36*), isolation of ESBL producers has not been reported. On the other hand, ceftriaxone-resistant *Salmonella* isolates, which produce plasmid-mediated AmpC-type β-lactamase such as CMY-2, are proliferating globally (*37*). Ceftriaxone-resistant *Salmonella* and *E. coli* strains have been also isolated from cattle recently in the United States (*23*,*32*,*33*,*35*). These findings suggest that cattle can serve as an incubator or reservoir of these antimicrobial drug–resistant bacteria. The authors of the U.S. studies suggested that the emergence of the AmpC-mediated cephalosporin resistance may have been a consequence of the use of ceftiofur, the only cephalosporin approved for systemic use in food animals in the United States (*23**,**32**,**35**)*. Dunne et al. support this hypothesis, indicating that the use of ceftiofur in cattle may have contributed to the emergence of the ceftriaxone-resistant *Salmonella* because the isolate shows cross-resistance between ceftiofur and ceftriaxone (*33*). In our study, all the isolates producing CTX-M-2 β-lactamase were also highly resistant to ceftiofur. What antimicrobial agents had been used at Japanese cattle farms where the CTX-M-2 producers were isolated is not well known, since the samples were collected at slaughterhouses. However, ceftiofur was the only expanded-spectrum cephalosporin approved for livestock in Japan when our study was conducted. In addition, the MIC (>1,024 μg/mL) of ceftiofur for CTX-M-2 producers isolated in this study was relatively higher than those (2 to >32 μg/mL) for TEM- or SHV-derived ESBL producers (*38*) that have been emerging in so many humans. Thus, the emergence of CTX-M-2 β-lactamase–producing *E. coli* in Japan might also be a consequence of the use of ceftiofur for livestock. However, why CMY-2 type class C β-lactamase is predominantly found in livestock in the United States is not clear. The types of antimicrobial agents and their use for livestock in that country may have contributed to its high prevalence of CMY-2 producers, although no statistical data are available about the differences in usage of antimicrobial agents between the United States and Japan. Continuous and prospective investigations of veterinary usage of the antimicrobial agents as well as surveillance of antimicrobial-resistance seem necessary for preventing the emergence and further proliferation of antimicrobial-resistant bacteria in livestock.

The CTX-M-2 producers were not considered to reflect a clonal expansion of an *E. coli* strain carrying *bla*_CTX-M-2_ because five distinct RAPD patterns and plasmid profiles were identified in the nine isolates. These findings suggest that stealthy plasmid-mediated dissemination of *bla*_CTX-M-2_ gene among *E. coli* strains might be under way with the continuous consumption of the third-generation cephalosporin for veterinary use. Conjugal transfer of R-plasmid might occur in the intestinal tract, which is the main habitat of ESBL producers (*17*,*39*). Both strains GS553 and GS554 were isolated from the same fecal sample and produced the same β-lactamase, but they were different in terms of RAPD analysis and plasmid profile. Frequencies of transfer of the isolates were high (Table 3). These results suggested that conjugal transfer of the R-plasmids also occurred in the intestinal tract of cattle. Therefore, the possibility of further transfer of the resistance profile of *E. coli* to expanded-spectrum cephalosporins to other pathogenic bacteria such as *Salmonella* spp. and diarrheagenic *E. coli* should not be ignored.

The isolates in this study did not correspond to the serotypes of pathogenic *E. coli*, and they did not possess the virulence factors assayed. However, lack of virulence factors might contribute to subclinical increase of healthy carriers of these strains and might promote their dissemination among both cattle and human. Especially in livestock, environmental contamination and transmission among individual animals by these strains could expand rapidly because of their breeding system. Therefore, CTX-M-2 producers may well be disseminated even further in cattle farms hereafter. Although nosocomial bacteria that produce extended-spectrum class A β-lactamases have thus far been considered to emerge only among in humans, our study suggested that CTX-M-2 producers could potentially emerge in livestock and that cattle might be an original reservoir of CTX-M-2 producers. Therefore, active and continuous surveillance and strategic countermeasures are necessary for antimicrobial-resistant bacteria, including those strains producing such β-lactamases as CTX-M-type, CMY-type (*37*,*40*) and metalloenzymes (*16*) in livestock, especially in countries where these producers have emerged in human populations.
